# Moist wound dressing and its application in distant skin flap in cats

**DOI:** 10.14202/vetworld.2021.734-738

**Published:** 2021-03-23

**Authors:** Erwin Erwin, Etriwati Etriwati, Rumi Sahara Zamzami, Cindy Trie Permatasari Hosea

**Affiliations:** 1 Laboratory of Clinic and Surgery, Faculty of Veterinary Medicine, Universitas Syiah Kuala, Banda Aceh, Indonesia; 2 Laboratory of Pathology, Faculty of Veterinary Medicine, Universitas Syiah Kuala, Banda Aceh, Indonesia; 3 Program Study of Veterinary Professional, Faculty of Veterinary Medicine, Universitas Syiah Kuala, Banda Aceh, Indonesia

**Keywords:** distant flap, moist dressing, objective, subjective

## Abstract

**Background and Aim:**

Wound healing is a dynamic and complex process that requires an appropriate environment to promote healing process. The healing of distant flaps in cats is determined by vascularization, nutrient sufficiency for the cells, and stability of skin flaps. This study aims to evaluate the healing of distant flaps treated with moist wound dressing through subjective and objective observation in five cats with wounds in the forelimb and hindlimb area to determine the time to cut the skin flaps from the donor site.

**Materials and Methods:**

In this study, five Indonesian local cats with wounds of various sizes in the limb were brought to the Veterinary Teaching Hospital. The sterile wound treatment included the administration of anesthesia, wound debridement, and distant flap closure in the thoracic and abdominal area. The distant flap and time to cut the skin flaps from the donor site were evaluated through subjective and objective examinations.

**Results:**

The subjective observation on the color of the distant skin flaps showed redness and response to pain on day 3 after surgery, whereas the objective observation, which was based on drug absorption capability and drug effect showed good results. On day 7 after surgery, the skin flaps from the donor site were cut and showed good progress.

**Conclusion:**

Overall, moist dressing helps in stabilizing the distant flap, allowing the distant flaps from the donor site to be cut on day 7 after surgery.

## Introduction

Wounds on the skin can be caused by injuries, burns, bites [1], chemical irritants, and tumor removal [2]. Skin wounds are usually treated by bringing the two wound edges close together, but if the wound is large in size, it is treated by making a skin flap [1]. If the wound is not treated by the skin flap method (the wound healed under the owner’s care), it creates scar tissue with a thickened skin like mass in the surgery region [3]. No general standard protocol for wound treatment has been established. However, the principle of adequate wound debridement care is accepted [4]. Skin flaps are classified based on anatomical vascularization, method of use, and tissue components [5]. The flap in which the skin originated from the area adjacent to the wound is called a local flap, whereas a flap in which the skin originated from the area away from the wound is called a distant flap [6].

Distant flap is used when no healthy skin tissue is present around the wound. It involves the movement of several tissues, including the skin, fascia, muscles, bones, or some combinations of these that become the donor site to another body part that needs them, namely, the recipient site [7]. Distant skin flap belongs to a category of skin flaps that need more complex care compared with other types of skin flaps. Animals will stand on their other three extremities during recovery, and it will try to pull the extremity treated with the distant flap. The long treatment duration causes the animal to feel uncomfortable and experience joint stiffness and leads to muscle atrophy [8].

Several types of wound dressing are available in the market today; among them are silver-containing hydrophilic fiber dressing, antibacterial dressing, and wet dressing [9]. These dressings have their own advantages and disadvantages in wound treatment. We often use these dressings in wound treatment, but the healing process of the distant flap method requires extensive work and remains a challenge for the veterinarian to apply it with the appropriate dressing. Thus, a high-quality and functional dressing will speed up the wound healing process, reduce the patient’s pain, and prevent the wound from germ infection [10,11].

This study aims to evaluate the healing of distant flaps treated with moist wound dressing through subjective and objective observation in five cats with wounds in the forelimb and hindlimb area to determine the time to cut the skin flaps from the donor site.

## Materials and Methods

### Ethical approval

All procedures performed in this research have been approved by the Veterinary Ethics Committee of the Faculty Veterinary Medicine, Universitas Syiah Kuala, Indonesia (certificate number 62/KEPH/II/2019).

### Study period, location and study design

During August to December 2019, the Veterinary Teaching Hospital, Faculty of Veterinary Medicine (Universitas Syiah Kuala, Banda Aceh, Indonesia) treated five cats of various ages with wounds in the limb. All cats underwent clinical, hematological, and blood chemical examinations to ensure that their physical condition was appropriate for surgery.

The cats had fasted for 8 h before surgery and were given premedication: Ketamine 10% (10 mg/kg BW) (Ketamil^®^, Troy Laboratories PTY Limited, Australia) and diazepam 0.05% (0.5 mg/kg BW) (Valisanbe^®^, Sanbe Farma, Indonesia) through intramuscular injection. For maintenance, an inhalation anesthetic (Isoflurane^®^, Piramal Critical Care, Inc., Bethlehem, PA 18017, USA imported by PT. Pratapa Nirmala, Tangerang, Indonesia) was administered during the surgery [12]. The cats were positioned in the lateral recumbent position and the wound edges were debrided to remove necrotic tissue and excessive granulation. The limb with wound was flexed to the thoracic area (for forelimb wound) and abdominal area (for hindlimb wound) to determine the skin incision location on the donor site (distant flap).

During the first surgery, skin flap incision (donor site) was performed accordingly to the size of the wound (recipient site) of each patient in the craniocaudal direction of the thoracic or abdominal area. The skin was unfolded, and then, a stitch was made on each corner of the skin flap to uplift the skin. The skin flap (donor site) was put on the cranial, proximal, and distal border edges of the wound (recipient site) and then sutured with silk surgical suture 3.0 USP (Silkam^®^, Romed Medical, Indonesia) in a simple interrupted pattern. The skin around the extremity was sutured to the skin around the donor site to reduce movement [13]. The distant flap area was dressed with moist dressing impregnated with framycetin sulfate (Sofra-Tulle^®^, Pantheon UK Limited, Swindon, UK, for Sanovi-Aventis, Thailand) for 12 days and supported with a flexible bandage. The dressings were replaced 4 times, that is, on days 3, 6, 9, and 12 [14]. After surgery, the wound was treated with amoxicillin and clavulanic acid (10 mg/kg BW) (Claneksi^®^, Sanbe Farma, Indonesia) and carprofen (2.2 mg/kg BW) (Rimadyl^®^, Pfizer/Zoetis, USA) for 7 days with an interval of 2 times a day. The second surgery is the cutting of the skin flap from the donor site area when the neovascularized tissue has grown and the flap could survive without vascularization from the donor site. The cut part of the flap was then sutured so the wound area would not be exposed, and the distant flap area was treated with moist dressing again. The sutures were removed when the wound has healed and both wound edges have closed. To determine the best time to cut the skin from the donor site on the recipient site area, the assessment of distant flap healing was done subjectively and objectively [14]:

a. Subjective parameters. The color of the distant skin flap (donor site) compared with the surrounding skin on post-surgical days 3, 6, 9, 12, and 18 for cutting the donor site on the second surgery. The categories for the assessment of skin color changes were four (black/necrosis), three (ischemia), two (hyperemia), and one (same as surrounding skin).
b. Pain response of the distant skin flap on post-surgical days 3, 6, 9, 12, and 18. The assessment of pain response was performed by pressing the donor site skin and categorized as three (no response/necrosis), two (responsive/pain), and one (no pain response/healed).
c. Observation of the hair growth presence on the surface of donor site skin after surgery.
a. Objective parameters. Injection of 0.2 mL of NaCl 0.9% subcutaneously on the donor site skin area on day 18 after the second surgery and evaluation of absorption time.
b. Injection of 0.2 mL adrenaline subcutaneously on the donor site skin area on day 18 after the second surgery and then observation of sympathetic nerve reaction as an effect of sympathomimetic stimuli.

### Cutting time

During the healing of the distant flaps, the cats can walk with three limbs, since one limb is flexed to the donor site to close the wound. When the skin from the donor site is taken well, the donor site skin can be cut. A good distant flap treatment will dramatically affect the required time to cut the donor site.

### Statistical analysis

The quantitative data from the skin color and pain response observation were analyzed using analysis of variance, whereas the time for hair growth, time for cutting the donor site (distant skin flaps), absorption, and reaction drug were analyzed through independent-sample t-test using SPSS version 24.0 (IBM Corp., NY, USA).

## Results

### The color change of the distant skin flap

**Figure-1:**
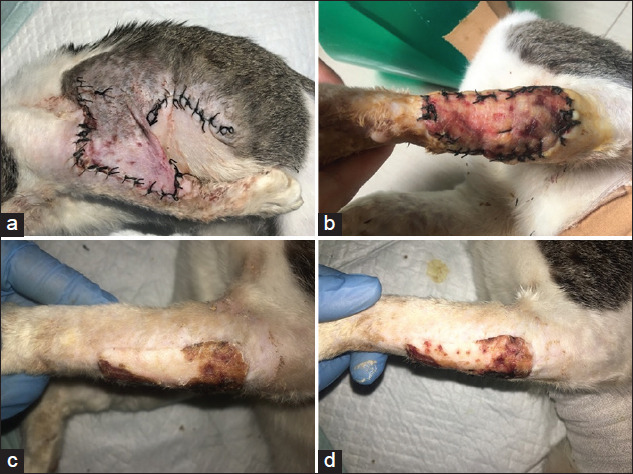
Skin donor figures during post-surgery treatment. (a) Skin donor condition on day 3; (b) skin donor condition on day 9 cutting of the skin flap from donor site area; (c) skin donor condition on day 12; and (d) skin donor condition on day 24 after distant flap surgery.(b-d) After cutting of the skin flap from donor site area.

A few days after treatment, the color change of the distant skin flap (donor site) showed good improvement, characterized by the color of the skin flap resembling the surrounding skin at the end of observation. On average, the color of the distant skin flaps (donor site) showed hyperemia until it resembles that of the surrounding skin (recipient site) with a significant difference in observation days (p<0.05). No significant difference was observed in the mean score for the color of the distant skin flaps (donor site) on day 3 (2.0±0.0) and day 6 (1.4±0.5) after the first surgery (p>0.05), whereas a significant difference (p<0.05) was observed on day 18 after the second surgery (Table-1). On days 9 and 12 after the second surgery to cut the distant skin flap from the thoracic and abdominal area (donor site), the mean score of the color of the donor site was 1.8±0.8 and 1.2±0.4, respectively. The application of moist dressing for distant skin flaps showed a good effect. On day 3, the distant skin flap showed hyperemia, which is a sign of good neovascularization growth, as shown in Figure-1. The wound edges of the distant skin flap (donor site) began to close and resemble the color of the surrounding skin on day 6. On days 12 and 18 after the second surgery, some parts of the cut donor skin underwent ischemia with no significant difference (p>0.05). A few days after the treatment of the distant flap with a moist dressing, the donor site began to show improvement. On day 18, the surface of the donor site had matched perfectly with the color of the surrounding skin.

**Table-1:** Observation of subjective distant skin flap color changes.

The cat	Ages (years)	Donor site area	Observation days				

			3	6	9	12	18
1	4	Thorax	2	1	2	1	1
2	2	Thorax	2	2	1	1	1
3	4	Thorax	2	1	2	1	1
4	3	Abdomen	2	2	3	2	1
5	3	Abdomen	2	1	1	1	1
Average±SD			(2.0±0.0)^ a^	(1.4±0.5)^ a, b, c, d^	(1.8±0.8)^ a, b, c^	(1.2±0.4)^ c, d^	(1.0±0.0)^ c^

The changes of skin color categorized into scoring: 4 (necrosis), 3 (ischemia), 2 (hyperemia), 1 (same as surrounding skin). Value with same superscripts in row (a-d) indicates not significant difference (p>0.05)

### Pain response

During treatment after surgery, the animals were calm and did not move a lot. Based on the pain response assessment on day 3 after surgery, the animals showed pain response when pressure was applied to the skin of the donor site area with a mean score of 2.0±0.0. On day 6, the pain response began to diminish with a score of 1.4±0.5, as presented in Table-2 with a significant difference in observation days (p<0.05). The donor site for the distant skin flaps was cut on average on day 7.2±0.4; therefore, observation on day 9 showed pain response due to inflammation (post-second surgery) with a score of 1.8±0.4. On days 12 and 18 after surgery, the skin of the distant skin flaps (donor site) no longer showed pain response and had healed completely with no significant difference (p>0.05).

**Table-2:** Observation of subjective pain response test.

The cat	Ages (years)	Donor site area	Observation days				

			3	6	9	12	18
1	4	Thorax	2	1	2	1	1
2	2	Thorax	2	2	1	1	1
3	4	Thorax	2	1	2	1	1
4	3	Abdomen	2	2	2	1	1
5	3	Abdomen	2	1	2	1	1
Average±SD			(2.0±0.0)^ a^	(1.4±0.5)^ b, c^	(1.8±0.4)^ a, b^	(1.0±0.0)^ c^	(1.0±0.0)^ c^

The pain test given a categorized scoring; 3 (no pain/necrosis), 2 (pain), 1 (no pain/healed). Value with same superscripts in row (a-d) indicates not significant difference (p>0.05)

### Observation of hair growth, time to cut donor site, absorption, and drug test reaction

Hair growth on the distant skin flap area (donor site) was clearly visible on day 6 after the first surgery. However, some fine short hair has started to become palpable on day 5 after the first surgery, as presented in Table-3, with no significant difference between the thoracic and abdominal donor site (p>0.05). The injection of 0.2 mL of NaCl 0.9% under the distant skin flaps (donor site) showed the time to be perfectly absorbed in 4 min, which is similar to other healthy skin tissue with no significant difference between the thoracic and abdominal donor site (p>0.05). Moreover, the injection of adrenaline under the distant skin flaps (donor site) showed the effect of pupil dilatation at 5 min, whereas the injection of adrenaline to the healthy skin tissue showed the effect at 4.5 min with no significant difference between the thoracic and abdominal donor site (p>0.05). The application of moist dressing for the treatment of distant skin flaps (donor site) resulted in moist wound condition and allowed neovascularization immediately, so as on average the distant skin flaps (donor site) could be cut on day 7.2±0.4 after the first surgery with no significant difference between the thoracic and abdominal donor site (p>0.05). Table-3 shows the mean values of the duration of the cutting of the distant skin flaps (donor site), absorbency time of NaCl 0.9%, and drug test reaction time of the distant skin flaps.

**Table-3:** Observation of hair growth, time to cut donor site, absorption, and reaction drug test.

The cat	Age (years)	Donor site area	The time needed for hair growth (days)	Time to cut donor site	The objective observation (min±SD)	

					Absorption drug	Reaction drug
1	4	Thorax	6^a^	7^a^	4.2^a^	5.1^a^
2	2	Thorax	6^a^	7^a^	4.5^a^	5.0^a^
3	4	Thorax	6^a^	7^a^	4.1^a^	4.3^a^
4	3	Abdomen	7^a^	7^a^	4.3^a^	5.2^a^
5	3	Abdomen	6^a^	8^a^	5.1^a^	5.5^a^
Average±SD			(6.2±0.4)	(7.2±0.4)	(4.4±0.4)	(5.0±0.4)

The observation of hair growth, time to cut donor site, absorption, and reaction drugs. Value with same superscripts in column (a-d) indicates not significant difference (p>0.05)

## Discussion

The healing process of distant skin flaps depends on the success of post-surgical treatments. In this study, three cats with forelimb wound received the distant flaps from the skin of the thoracic area, whereas two cats with hindlimb wound received the skin from the donor site of the abdominal area. The surgical technique of distant skin flap requires donor site skin with sufficient vascularization to support the survival of the skin flap when the skin is removed from the donor site. Distant flaps are mainly used to close the wounds of the distal surface of the lateral forelimb and hindlimb [15]. The lateral thoracic and abdominal areas were chosen as the donor site because of the availability of loose skin [16].

The wound bed (recipient site) should be free of necrotic tissue and contamination and have a reddish healthy granulation tissue. Aseptic surgical procedure made it necessary for the manipulation of atraumatic tissue to minimize infection and preserve the blood supply on the wound area [17]. The redness on the skin flaps showed that the flap has fused, has good neovascularization, and can survive without vascularization from the donor site [1]. The application of moist dressing containing antibiotics reduces the risk of infection and maintains the wound in a moist condition. A moist condition will accelerate neovascularization, granulation, and epithelization [8]. Good vascularization also facilitates the mobilization of inflammatory cells and fibrins to the wound area, therefore determining the success of the distant flap. A surgical wound causes clinical pain due to changes in the nervous system’s sensitivity to pain stimuli. The pain will subside as the inflammatory reaction begins to diminish and the tissue heals. Clinical pain belongs to pain caused by sensory nervous system reaction to certain stimuli, that is, a body stimuli signal [6].

Hair growth indicates the success of the flap procedure. It is consistent with the donor site and may be a little different than the surrounding skin but still shows a better appearance. Flaps with donor site skin from the thoracic area resulted in proper skin and hair for the recipient site [16]. Good neovascularization characterized by redness on the donor site indicates that the donor site can be cut. After it is cut, the donor site will no longer receive a nutrition supply from the vascularization of the original flap. The neovascularization formed between the recipient site and donor site becomes the source of nutrition supply for the donor site until it perfectly merges with the recipient site. Good neovascularization in the donor site accelerates the absorption time of drugs administered [14]. In clinical practice, the administration of drugs on the skin is greatly determined by the function of the stratum corneum, although bioactive substances of the drug also affect absorption time [18].

## Conclusion

Overall, based on the subjective and objective evaluation of distant flaps using moist dressing, the distant flaps that were treated with moist dressing showed good development regarding the redness of the skin flaps, non-necrosis tissue, hair growth, and ability to absorb and distribute the drugs. Thus, the distant flaps can be cut on the 7^th^ day after the first surgery.

## Authors’ Contributions

EE, EEt, and RSZ conceived and designed the anesthesia and surgery. EE and CTPH executed the postsurgical treatment, analyzed, and interpretation of distant flaps healing. All authors interpreted and critically revised the manuscript for important intellectual contents and approved the final version.
